# Chemical Constituent Analysis of *Ranunculus sceleratus* L. Using Ultra-High-Performance Liquid Chromatography Coupled with Quadrupole-Orbitrap High-Resolution Mass Spectrometry

**DOI:** 10.3390/molecules27103299

**Published:** 2022-05-20

**Authors:** Shanshan Cao, Min Hu, Lingli Yang, Meiqin Li, Zhen Shi, Wenming Cheng, Yazhong Zhang, Fei Chen, Sheng Wang, Qunlin Zhang

**Affiliations:** 1 School of Pharmacy, Anhui Medical University, Hefei 230032, China; 15555455280@163.com (S.C.); minhu3597@163.com (M.H.); 15850658857@163.com (L.Y.); lmq2860735640@163.com (M.L.); 18269712818@163.com (Z.S.); 17555801160@139.com (F.C.); 2 Anhui Institute for Food and Drug Control, Hefei 230051, China; 13956985695@139.com; 3 The Center for Scientific Research of Anhui Medical University, Hefei 230032, China; wangsheng_cpu@163.com

**Keywords:** *Ranunculus sceleratus* L. (RS), UHPLC-Q-Orbitrap HRMS, fragmentation pathway, chemical constituent

## Abstract

*Ranunculus sceleratus* L.(RS) has shown various pharmacological effects in traditional Chinese medicine. In our previous study, the positive therapeutic effect on α-naphthylisothiocyanate induced intrahepatic cholestasis in rats was obtained using TianJiu treatment with fresh RS. However, the chemical profile of RS has not been clearly clarified, which impedes the research progress on the therapeutic effect of RS. Herein, an ultra-high performance liquid chromatography coupled with quadrupole Orbitrap high-resolution mass spectrometry (UHPLC-Q-Orbitrap HRMS) method was developed to rapidly separate and identify multiple constituents in the 80% methanol extract of RS. A total of sixty-nine compounds (19 flavonoids, 22 organic acids, 6 coumarins, 4 lignans, 14 nitrogenous compounds, and 4 anthraquinones) were successfully characterized. A total of 12 of these compounds were unambiguously identified by standard samples. Their mass spectrometric fragmentation pathways were investigated. It is worth noting that flavonoids and lignans were identified for the first time in RS. In this study, we successfully provide the first comprehensive report on identifying major chemical constituents in RS by UHPLC-Q-Orbitrap HRMS. The obtained results enrich the RS chemical profile, paving the way for further phytochemical study, quality control, and pharmacological investigation of RS.

## 1. Introduction

*Ranunculus sceleratus* L. (RS), an annual herbaceous plant belonging to the *Ranunculus* L. family, has been listed among the top herbs in the Shennong Traditional Herbal Scriptures, written in the Western Han Dynasty. Compendium of Materia Medica notes that fresh RS can be pasted onto the acupuncture point of Cunkou overnight, (hyperemia and blistering in the skin), to cure jaundice induced by malaria. TianJiu treatment with fresh RS patches exhibited a positive therapeutic effect on α-naphthylisothiocyanate-intrahepatic cholestasis in rats by pasting on the acupuncture points of Dazhui, Ganshu (both sides), and Jizhong in our previous study [1]. The protoanemonin of fresh RS is commonly used as a robust blistering agent. It has been reported that protoanemonin is poisonous, but toxins will be destroyed when fresh RS is heated or dried. Moreover, the anti-inflammatory activity of the extract of RS administered orally at a dose of 100 mg·kg^−1^ was obtained in Wistar rats by inhibiting the induced hind paw edema [2]. A dosage between 3 and 9 g is recorded in Zhonghuabencao when taken orally as a decoct soup to clear heat, reduce swelling, disperse the knot, and relieve pain. However, little is known about the chemical basis of the therapeutic effect of RS. Over the past few years, several chemical constituents have been isolated from RS, such as emodin, scoparone, isoscopoletin, protocatechualdehyde, protocatechuic acid, hexadecanoic acid, β-sitosterol, stigmast-4-ene-3,6-dione, stigmasterol, 1-docosene, and stigmast-5-en-3-ol [3,4]. In our previous study, 31 volatile compounds were identified in fresh RS by GC-MS analysis. The main chemical constituents were lactones and phenolic acids, including protoanemonin, 2,5-furandione, 2-propanedioic acid, and phenacetic acid [1]. Considering the positive therapeutic application of RS, it is of great importance to establish a rapid and reliable method for chemical profiling RS.

Ultra-high performance liquid chromatography coupled with quadrupole Orbitrap high-resolution mass spectrometry (UHPLC-Q-Orbitrap HRMS), with mass accuracy and high sensitivity for precursor and product ions, exhibits high resolution. Conventional separation and identification processes are time and plant-material-consuming. In contrast, the UHPLC-Q-Orbitrap HRMS method has shown high efficiency in the separation, identification, and analysis of multiple chemical constituents of traditional Chinese medicines (TCMs) [5,6,7]. Therefore, this method can tentatively identify components without reference standards according to exact MS data. The UHPLC-Q-Orbitrap MS method combined the inclusion list, and data-dependent acquisition was established to achieve a comprehensive characterization of the TCM formula Kai-Xin-San, and 211 compounds were identified by comparison with reference standards, literature data, and databases [8]. A total of 18 phenolic acids were identified in *Lycium ruthenicum* Murray by comparing the retention time and the exact *m*/*z* in authentic standards using UPLC-Q-Orbitrap MS [9].

To the best of our knowledge, the comprehensive identification of multiple chemical constituents in RS has not been reported. A series of studies were carried out for the rapid characterization of the chemical profile in RS. First, we established a chemical database for the compounds of *Ranunculus* L. family plants and collected as many standards as possible. Second, samples were analyzed by UHPLC-Q-Orbitrap HRMS technology for full-spectrum scanning, and the spectrum information was obtained by Xcalibur 4.1 software. Finally, after characterizing the diagnostic ions and fragmentation rules for the standards, the fragment information in the mass spectrum was compared with the literature to confirm the chemical constituents of RS. In the present study, 19 flavonoids, 22 organic acids, 6 coumarins, 4 lignans, 14 nitrogenous compounds, and 4 anthraquinones from RS were identified by UHPLC-Q-Orbitrap HRMS, providing in-depth knowledge of its chemical constituents and offering valuable information regarding its quality control and further pharmacological study.

## 2. Results and Discussion

### 2.1. Optimization of UHPLC-Q-Orbitrap HRMS Conditions

The composition of the UHPLC mobile phase should be systemically optimized to achieve the best chromatographic and mass spectrometric properties for separation and analysis. To assess the resulting peak shape and signal strength, different mobile systems that consisted of methanol−0.1% formic acid aqueous solution or acetonitrile−0.1% formic acid aqueous solution were investigated. In the result, acetonitrile−0.1% formic acid aqueous solution was chosen as the UHPLC mobile phase for gradient elution. In terms of sample processing, 60%, 80%, and 100% methanol (*v*/*v*) were examined to obtain the optimal RS extract that contains the more chemical constituents of RS. It was found that 80% methanol showed the best extraction efficiency based on the number of peaks and was selected as the extraction solvent for RS. An 80% methanol extract of RS was then analyzed in the positive and negative ion modes. Most chemical constituents showed higher responses in negative ionization mode than in positive ionization mode. In addition, to improve the sensitivity and accuracy, the ion source voltage and capillary temperature were optimized. The optimal conditions are described in Section 3.3.

### 2.2. Identification of Chemical Constituents in RS

**Figure 1:**
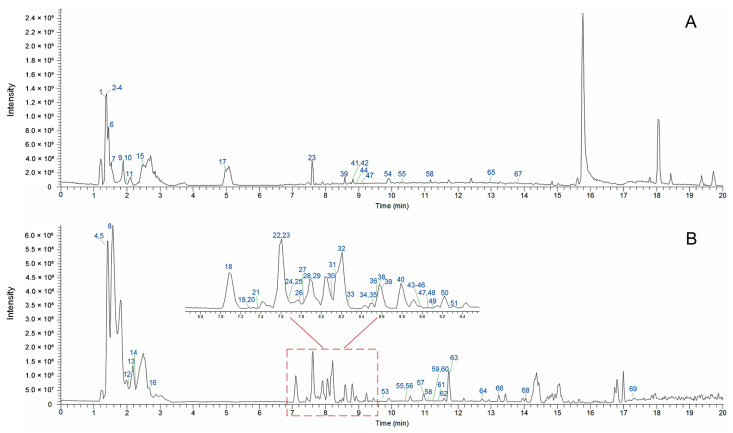
Base peak chromatograms of RS in positive ionization mode (**A**) and negative ionization mode (**B**).

Under the optimal UHPLC-Q-Orbitrap HRMS conditions, the base peak chromatograms obtained in positive and negative ionization modes for the 80% methanol extract of RS are shown in Figure 1. As shown in Table 1, a total of 69 compounds (19 flavonoids, 22 organic acids, 6 coumarins, 4 lignans, 14 nitrogenous compounds, and 4 anthraquinones) in the 80% methanol extract of RS were either unambiguously identified (12 compounds) or tentatively characterized (57 compounds). Reference standards of isoscopoletin, scopoletin, and scoparone were detected in positive ionization mode (Figure S1), and reference standards of aesculetin, quercetin, protocatechuic acid, salicylic acid, ferulic acid, luteolin, caffeic acid, emodin, and oleanic acid were detected in negative ionization mode (Figure S2). Next, the names and chemical structures of the other 57 compounds were preliminarily inferred by comparing MS ion fragmentation information with relevant literature. Altogether, the chemical structures of identified 69 compounds are summarized in Figure S3.

#### 2.2.1. Flavonoids

**Figure 2:**
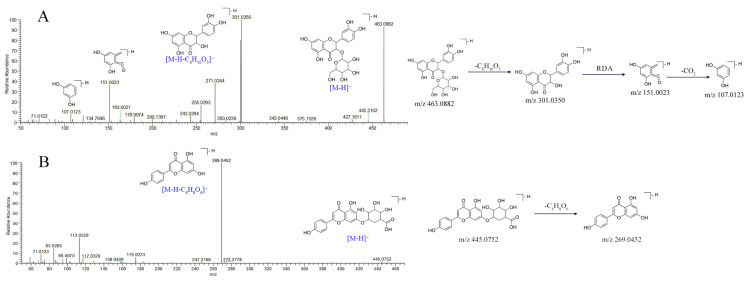
Characteristic of MS/MS spectra and possible fragmentation pathways of hyperoside (**A**) and apigenin-7-O-glucuronide (**B**).

Flavonoids refer to a class of naturally occurring bioactive compounds in herbal medicine. The principal mass spectrometric fragmentation mechanisms for flavonoids are loss of neutral fragments, such as H_2_O, CH_3_, CO, CO_2_, and the cleavage of retro-Diels-Alder (RDA) at glycosyl bonds. The product ion spectrum of compound **39** shown in Figure 2A is representative. In ESI^−^, compound **39** produced the deprotonated molecular ion at *m*/*z* 463.0882 [M − H]^−^, which could form the fragment ion *m*/*z* 301.0350 [M − H − C_6_H_10_O_5_]^−^ by losing one glucose sugar group (C_6_H_10_O_5_). After undertaking the RDA reaction, a series of product ions could be produced, such as *m*/*z* 151.0023 and 107.0123. By comparing their molecular formulas and fragmentation patterns with those reported in the literature [29], compound **39** was tentatively identified as hyperoside, reported here for the first time in RS. Similarly, the [M − H]^−^ ion of compound **47** was shown at *m*/*z* 445.0752, and was tentatively identified as apigenin-7-O-glucuronide. Characteristic fragment ions *m*/*z* 269.0452 were produced by successive loss of glucuronide [28]. Glucuronide residues at *m*/*z* 175.0233 and *m/z* 113.0229 could always be observed in glycosides. The mass spectra and proposed major fragmentations with structures are shown in Figure 2B. Except for luteolin (**55**) and quercetin (**56**), confirmed by reference standards, all other flavonoids were identified similarly. The other flavonoids identified according to their molecular mass, formulas, MS/MS fragments, and related literature studies, including kaempferol-3,7-di-O-glucoside (**26**), kaempferol-O-sophoroside (**30**), quercetin-O-(pentosyl) hexoside isomer (**33**), isoorientin (**35**), isorhamnetin (**41**), kaempferol (**42**), isorhamnetin-3-O-glucoside isomer (**43**), luteoloside (**45**), luteolin-7-O-glucuronide (**48**), apigenin (**58**), chrysoeriol (**59**), diosmetin (**61**), rhamnetin (**62**), pinocembrin (**64**), 7,3’-dihydroxy-8,4’-dimethoxyisoflavone/isomer (**65**). Flavonoids were identified for the first time in RS, and pharmacological studies have demonstrated that flavonoids have good efficacy in treating cholestatic liver disease [39].

#### 2.2.2. Organic Acids

**Figure 3:**
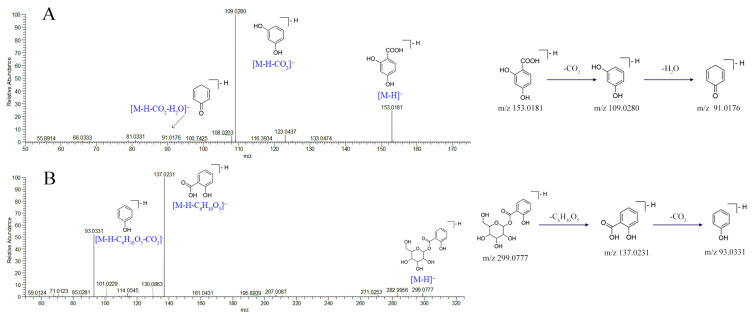
Characteristic of MS/MS spectra and possible fragmentation pathways of protocatechuic acid (**A**) and salicylic acid-O-glucopyranoside (**B**).

Organic acids widely occur in natural plants, especially in herbs. In negative ionization mode, organic acids exhibited deprotonated molecular peaks and easily lost CO_2_ and CO to generate corresponding fragment ion peaks. Moreover, the losses of small molecules (like H_2_O) or radicals (like CH_3_) sometimes occurred. For instance, protocatechuic acid (compound **21**) showed a molecular formula of C_7_H_6_O_4_ and a deprotonated molecule [M − H]^−^ peak at *m*/*z* 153.0181. The deprotonated molecule lost a CO_2_ moiety to form a fragment ion [M − H − CO_2_]^−^ at *m*/*z* 109.0280. Then, it was dehydrated to form the [M − H − CO_2_ − H_2_O]^−^ fragment ion of *m*/*z* 91.0176. The fragmentation pathways of compound **21** are shown in Figure 3A. Compound **21** was unambiguously identified as protocatechuic acid by comparing its MS/MS fragmentation pattern and retention time of reference standard. Likewise, compounds **32**, **46**, **53**, and **69** were identified as caffeic acid, ferulic acid, salicylic acid, and oleanic acid by reference standards. The deprotonated molecule [M − H]^−^ of compound **25** peak was at *m*/*z* 299.0777, and its chemical structure and fragmentation pathway are shown in Figure 3B. Compound **25** was identified as salicylic acid-O-glucopyranoside according to the literature [19]. The other compounds were identified according to their molecular mass, formulas, MS/MS fragments, and related literature studies, including gluconic acid (**5**), malic acid (**8**), aconitic acid (**12**), furoic acid (**13**), citric acid (**14**), succinic acid (**16**), protocatechuic acid-O-glucose (**18**), vanillic acid-O-glucopyranoside (**19**), hydroxytyrosol-1-glucopyranoside (**20**), caffeic acid-O-glucopyranoside (**22**), protocatechualdehyde (**27**), *p*-coumaric acid-O-glucopyranoside (**29**), suberic acid (**36**), coumaric acid (**40**), azelaic acid (**50**), and *p*-hydroxybenzene propanoic acid (**51**).

#### 2.2.3. Coumarins and Lignans

**Figure 4:**
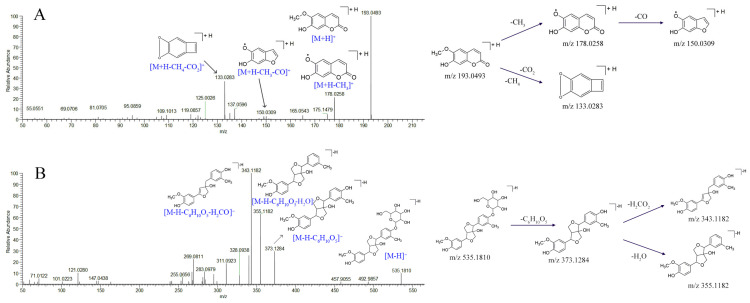
Characteristic of MS/MS spectra and possible fragmentation pathways of scopoletin (**A**) and hydroxylpinoresinol 4′-O-glucopyranoside (**B**).

Six coumarins were identified from the 80% methanol extract of RS, including esculin (**24**), scopolin (**28**), aesculetin (**31**), isoscopoletin (**37**), scopoletin (**44**), and scoparone (**54**). Their fragmentation patterns in mass spectrometry were investigated, and neutral losses of CO and CO_2_ could be commonly observed. A typical coumarin, scopoletin (**44**), was taken as an example to investigate the MS/MS fragmentation pattern of coumarin in RS. The protonated molecular ion of compound **44** was *m*/*z* 193.0493 [M + H]^+^ in positive ESI mode. Scopoletin produced a fragment ion [M + H − CH_3_]^+^ at *m*/*z* 178.0258 by demethylation, which further lost one CO moiety, and the fragment ions of [M + H − CH_3_ − CO]^+^ at *m*/*z* 150.0309 were generated. Scopoletin also directly lost one CH_4_ and CO_2_ neutral moiety to generate a product ion [M + H −CH_4_ − CO_2_]^+^ at *m*/*z* 133.0283, indicating the C-6 methoxy substituents and lactone structures. The possible fragmentation pathway for scopoletin is proposed in Figure 4A. The esculin (**24**) and scopolin (**28**) were identified according to related literature studies [20,23]. Compounds **31**, **37**, **44,** and **54** were confirmed by comparison with the available reference standards.

Lignans are a class of natural compounds synthesized by polymerizing two phenylpropanoid derivatives (C6-C3 monomers). Compounds **38**, **49**, **52**, and **57** were furofuran-type lignans. Taking Compound **38** as an example, the deprotonated molecular ion *m*/*z* 535.1772 was detected in the spectrum. Its MS/MS fragment ions at *m*/*z* 373.1284 [M − H − C_6_H_10_O_5_]^−^, *m*/*z* 355.1182 [M − H − C_6_H_10_O_5_ − H_2_O]^−^, and *m*/*z* 343.1182 [M − H − C_6_H_10_O_5_ − H_2_CO]^−^ were observed in negative ionization mode. Compound **38** was identified as 1-hydroxylpinoresinol 4′-O-glucopyranoside according to the literature [19]. The possible fragmentation mechanism of compound **38** is depicted in Figure 4B. Compounds **49**, **52,** and **57** were tentatively identified as matairesinoside, 1-hydroxypinoresinol, and matairesinol according to their MS/MS fragments and related literature studies [19].

#### 2.2.4. Nitrogenous Compounds

**Figure 5:**
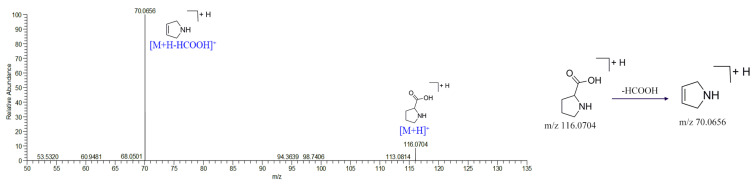
Characteristic of MS/MS spectra and possible fragmentation pathways of proline.

Amino acids, nucleobases, and other nitrogenous compounds respond strongly in positive ionization mode, and most of the second mass spectra are broken in the center of N^+^. In the present study, 3 nucleobases, 7 amino acids, and other nitrogenous compounds in RS were characterized. The usual fragmentation pathways, including the losses of NH_3_, H_2_O, and HCOOH, were observed in these compounds. Compound **6** was identified as proline based on short retention time and specific fragments. It has a pseudomolecular ion of *m*/*z* 116.0704, indicative of the molecular formula C_5_H_9_NO_2_. The fragment ion at *m*/*z* 70.0656 for [M + H − HCOOH]^+^ agrees with the literature studies due to losing one carboxyl group [11]. The chemical structures and fragmentation pathways are shown in Figure 5. In this way, other amino acids can be successfully characterized according to related literature studies, including asparagine (**1**), glutamic acid (**2**), threonine (**3**), pyroglutamic acid (**4**), phenylalanine (**17**), and tryptophan (**23**). In addition, nucleobases (compounds **7**, **9,** and **15**) and other nitrogenous compounds (compounds **10**, **11**, **34,** and **67**) were also detected and tentatively identified based on databases and the literature.

#### 2.2.5. Anthraquinonoids

**Figure 6:**
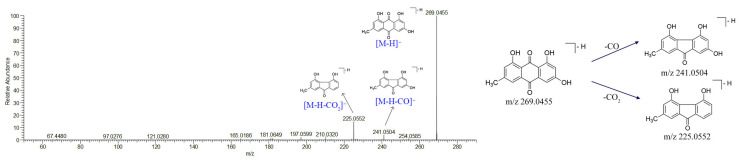
Characteristic of MS/MS spectra and possible fragmentation pathways of emodin.

By comparing the retention time and MS spectrum with authentic standards, compound **68** was identified as emodin. The protonated molecular ion of emodin was *m*/*z* 269.0455 [M − H]^−^ in negative ionization modes. Its MS/MS fragment ions were at *m*/*z* 241.0504 [M − H − CO]^−^ and *m*/*z* 225.0552 [M − H − CO_2_]^−^. The chemical structures and fragmentation pathways are shown in Figure 6. In addition, compounds **60**, **63,** and **66** were tentatively identified as 7-hydroxy-emodin, 1-O-methyl-emodin, and physcion according to their MS/MS fragments and related literature studies [34].

## 3. Materials and Methods

### 3.1. Chemicals and Reagents

High-performance liquid chromatography (HPLC)-grade acetonitrile and formic acid were purchased from Sigma (Sigma Aldrich, St. Louis, MO, USA). Analytical-grade methanol was purchased from Chinasun Specialty Products Co., Ltd. (Jiangsu, China). The aesculetin (Batch No. 5483, 99%), isoscopoletin (Batch No. 3620, 98%), scopoletin (Batch No. 5257, 98%), quercetin (Batch No. 1115, 98%), scoparone (Batch No. 1902, 98%), protocatechuic acid (Batch No. 5809, 99%), salicylic acid (Batch No. 5328, 99%), caffeic acid (Batch No. 2681, 98%), ferulic acid (Batch No. 8042, 99%), and emodin (Batch No. 8171, 98%) were purchased from Shanghai Standard Technology Co., Ltd. (Shanghai, China). Standards of luteolin (Batch No. C11352540, 98%) and oleanic acid (Batch No. M180850633, 98%) were obtained from Shanghai Macklin Biochemical Co., Ltd. (Shanghai, China). Ultrapure water (18.2 MΩ·cm^−1^) was purified by a Millipore system (Millipore Corp., Burlington, MA, USA).

The batch of *Ranunculus sceleratus* L. was collected in the Xinhe community of Feidong County (Hefei, China) and authenticated by Professor Huasheng Peng (School of Pharmacy, Anhui University of Chinese Medicine). Voucher specimens (Batch No. 20210401) were deposited in the herbarium of the School of Pharmacy, Anhui Medical University (Hefei, China).

### 3.2. Preparation of Sample and Standard Solutions

An aliquot of 0.1 g fine powder (<65 mesh) of RS samples was accurately weighed and placed in a 50 mL of a conical flask, and then 10 mL of 80% methanol−water (*v*/*v*) was added into the conical flask. After sonication for 30 min, the sample solution was cooled to room temperature. All the standards of aesculetin, isoscopoletin, scopoletin, quercetin, scoparone, protocatechuic acid, salicylic acid, ferulic acid, luteolin, caffeic acid, emodin and oleanic acid were dissolved in 80% methanol−water (*v*/*v*) at a concentration of 10 µg·mL^−1^ to prepare standard solutions. All the solutions were filtered through a 0.22 μm filter membrane (Bandao Corp., Shanghai, China) before analysis.

### 3.3. UHPLC-Q-Orbitrap HRMS System and Conditions

A UHPLC Dionex Ultimate 3000 (Thermo Scientific, San Jose, CA, USA) equipped with a cooling autosampler and column oven was utilized. The separation was performed on a Shim-pack GISS UHPLC C_18_ column (100 mm × 2.1 mm, 1.9 μm) (Shimadzu, Japan) with a column temperature maintained at 30 °C at a flow rate of 0.2 mL·min^−1^. Binary mobile solvents consisted of acetonitrile (A) and water containing 0.1% formic acid (B), and the following gradient elution program was used: 0−3 min, 2% A; 3–5 min, 2–30% A; 5–12 min, 30–70% A; 12–14 min, 70–95% A; 14–18 min, 95% A; 18–20 min, 95–2% A. The injection volume was set at 2 µL.

A Q-Exactive plus hybrid quadrupole-orbitrap mass spectrometer (Thermo Fisher Scientific, San Jose, CA, USA) with heat electrospray ionization (HESI) was employed. The mass conditions were set as follows: capillary temperature, 320 °C; auxiliary gas heater temperature, 200 °C; spray voltage, 4 kV/3.5 kV (positive/negative); Apex trigger, 2–6 s; Loop count, 5; S-lens RF level, 50 V. Full MS/dd-MS^2^ scan mode conditions were set as follows: Scan range, 75–1125 *m*/*z*; Full MS resolution, 70,000; dd-MS^2^ resolution, 17,500; Maximum injection time (IT), 50 ms; Isolation window, 1.0 *m*/*z*; Normalized collision energy (NCE), 20/40/60 eV; Automatic gain control (AGC) target, 1.0 × 10^5^; Dynamic exclusion, 10 s. Nitrogen was used for spray stabilization, for collision-induced dissociation experiments in the HCD cell, and as the damping gas in the C-trap.

### 3.4. Data Processing and Analysis

Tune 2.9 (Thermo Fisher Scientific, San Jose, CA, USA) was used to control the mass spectrometer, and Xcalibur 4.1 software (Thermo Fisher Scientific, San Jose, CA, USA) was used to control the instrument for data acquisition and analysis. The mass tolerance of MS and MS^2^ was within 5 ppm. The chemical formulas for all parent and fragment ions were calculated according to the exact mass, and the parameters are set as follows: C (0–60), H (0–120), O (0-60), and N (0–10).

## 4. Conclusions

The inherent variety of natural products in TCM has presented a big challenge in separation and detection techniques for the rapid characterization of its chemical profiling. In the present study, the chemical constituents of RS extract were determined by UHPLC-Q-Orbitrap HRMS. A total of 69 compounds, including 19 flavonoids, 22 organic acids, 6 coumarins, 4 lignans, 14 nitrogenous compounds, and 4 anthraquinones, were identified based on the comparison of their accurate masses, fragment ions, literature studies, and standard samples. Isoscopoletin, scopoletin, scoparone, aesculetin, quercetin, protocatechuic, salicylic acid, ferulic acid, luteolin, caffeic acid, emodin and oleanic acid were identified by standard samples. It is worth noting that flavonoids and lignans were identified for the first time in RS. This work can provide an essential chemical basis for quality control and further studies on the pharmacological and clinical application of RS.

## Figures and Tables

**Table 1:** 69 compounds identified in the 80% methanol extract of RS and their UHPLC-Q-Orbitrap HRMS data.

NO.	Retention Time (min)	Ionization Mode	Molecular Formula	Theoretical (*m*/*z*)	Measured (*m*/*z*)	Error (ppm)	Identified Compounds	MS/MS Fragments (*m*/*z*)	Ref.
**1**	1.37	[M + H]^+^	C_4_H_8_N_2_O_3_	133.0607	133.0604	−2.25	Asparagine	133.0604, 115.0391, 97.0286, 87.0558	[10]
**2**	1.40	[M + H]^+^	C_5_H_9_NO_4_	148.0604	148.0598	−4.05	Glutamic acid	148.0598, 102.0550, 84.0447	[11]
**3**	1.40	[M + H]^+^	C_4_H_9_NO_3_	120.0655	120.0655	0.00	Threonine	120.0655, 102.0553, 74.0607, 56.0503	[11]
**4**	1.40	[M + H]^+^	C_5_H_7_NO_3_	130.0498	130.0499	0.77	Pyroglutamic acid	130.0499, 102.0551, 84.0449	[11]
	1.40	[M − H]^−^	C_5_H_7_NO_3_	128.0342	128.0340	−1.56	Pyroglutamic acid	128.0339, 85.0279, 68.1163	
**5**	1.42	[M − H]^−^	C_6_H_12_O_7_	195.0499	195.0501	1.03	Gluconic acid	195.0501, 129.018, 75.0072	[12]
**6**	1.47	[M + H]^+^	C_5_H_9_NO_2_	116.0706	116.0704	−1.72	Proline	116.0704, 70.0656	[11]
**7**	1.51	[M + H]^+^	C_4_H_5_N_3_O	112.0505	112.0507	1.78	Cytosine	112.0507, 95.0244, 69.0455	[13]
**8**	1.57	[M − H]^−^	C_4_H_6_O_5_	133.0131	133.0129	−1.50	Malic acid	133.0129, 115.0023, 71.0124	[14]
**9**	1.91	[M + H]^+^	C_5_H_5_N_5_O	152.0567	152.0565	−1.32	Guanine	152.0565, 135.0300, 110.0351	[13]
**10**	1.91	[M + H]^+^	C_6_H_5_NO_2_	124.0393	124.0394	0.81	Nicotinic acid	124.0394, 106.0291, 96.0448, 80.0501	[15]
**11**	1.99	[M + H]^+^	C_6_H_6_N_2_O	123.0553	123.0554	0.81	Nicotinamide	123.0554, 80.0501	[16]
**12**	2.13	[M − H]^−^	C_6_H_6_O_6_	173.0080	173.0082	1.16	Aconitic acid	173.0082, 129.0179, 111.0072, 85.0279	[14]
**13**	2.17	[M − H]^−^	C_5_H_4_O_3_	111.0076	111.0073	−2.70	Furoic acid	111.0073, 83.0122, 67.0174	[17]
**14**	2.24	[M − H]^−^	C_6_H_8_O_7_	191.0186	191.0187	0.52	Citric acid	191.0187, 111.0073, 87.0072	[14]
**15**	2.62	[M + H]^+^	C_4_H_4_N_2_O_2_	113.0345	113.0347	1.77	Uracil	113.0347, 96.0084, 70.0294	[11]
**16**	2.64	[M − H]^−^	C_4_H_6_O_4_	117.0182	117.0179	−2.56	Succinic acid	117.0179, 99.0073, 73.0280	[15]
**17**	5.05	[M + H]^+^	C_9_H_11_NO_2_	166.0862	166.0861	−0.60	Phenylalanine	166.0861, 120.0809, 103.0545	[11]
**18**	7.09	[M − H]^−^	C_13_H_16_O_9_	315.0711	315.0722	3.49	Protocatechuic acid-O-glucose	315.0722, 153.0545, 109.0280	[18]
**19**	7.28	[M − H]^−^	C_14_H_18_O_9_	329.0867	329.0858	−2.73	Vanillic acid-O-glucopyranoside	329.0858, 167.033, 123.0437	[19]
**20**	7.28	[M − H]^−^	C_14_H_20_O_8_	315.1074	315.1092	5.71	Hydroxytyrosol-1-glucopyranoside	315.1092, 153.0545	[19]
**21**	7.37	[M−H]^−^	C_7_H_6_O_4_	153.0182	153.0181	−0.65	Protocatechuic acid	153.0181, 123.0437, 109.0280, 91.0176	Standard
**22**	7.61	[M−H]^−^	C_15_H_18_O_9_	341.0867	341.0873	1.76	Caffeic acid-O-glucopyranoside	341.0873, 179.0339, 135.0439	[19]
**23**	7.61	[M + H]^+^	C_11_H_12_N_2_O_2_	205.0972	205.0970	−0.98	Tryptophan	205.0970, 188.0703, 159.0914, 146.0598	[11]
	7.61	[M − H]^−^	C_11_H_12_N_2_O_2_	203.0815	203.0818	1.48	Tryptophan	203.0818, 186.0551, 159.091, 142.0649	
**24**	7.65	[M − H]^−^	C_15_H_16_O_9_	339.0711	339.0718	2.06	Esculin	339.0718,177.0183	[20]
**25**	7.67	[M − H]^−^	C_13_H_16_O_8_	299.0761	299.0777	5.35	Salicylic acid-O-glucopyranoside	299.0777, 137.0231, 93.0331	[19]
**26**	7.78	[M − H]^−^	C_27_H_30_O_16_	609.1450	609.1464	2.30	Kaempferol-3,7-di-O-glucoside	609.1464, 447.0930, 285.0402, 255.0295, 151.0023	[21]
**27**	7.82	[M − H]^−^	C_7_H_6_O_3_	137.0233	137.0231	−1.46	Protocatechualdehyde	137.0231, 109.0279, 108.0201, 93.0330	[22]
**28**	7.89	[M − H]^−^	C_16_H_18_O_9_	353.0867	353.0865	−0.57	Scopolin	353.0865, 191.0553, 179.0341, 173.0450, 135.0439	[23]
**29**	7.91	[M − H]^−^	C_15_H_18_O_8_	325.0918	325.0928	3.08	*p*-Coumaric acid- O-glucopyranoside	325.0928, 163.0388, 119.0487,	[19]
**30**	8.12	[M − H]^−^	C_27_H_30_O_16_	609.1450	609.1453	0.49	Kaempferol-O-sophoroside	609.1453, 429.0818, 284.0323, 255.0295, 227.0343	[24]
**31**	8.16	[M − H]^−^	C_9_H_6_O_4_	177.0182	177.0182	0.00	Aesculetin	177.0182, 149.0231, 133.0280, 121.0280	Standard
**32**	8.21	[M − H]^−^	C_9_H_8_O_4_	179.0339	179.0342	1.68	Caffeic acid	179.0342, 135.0437, 90.9966	Standard
**33**	8.25	[M − H]^−^	C_26_H_28_O_16_	595.1294	595.1301	1.18	Quercetin-O-(pentosyl)hexoside isomer	595.1301, 300.0274, 271.0248, 255.0294, 151.0022, 135.0433	[25]
**34**	8.51	[M − H]^−^	C_11_H_13_NO_3_	206.0811	206.0813	0.97	*n*-Acetyl-L-phenylalanine	206.0813, 164.0705, 147.0438, 118.9914, 91.0536	[26]
**35**	8.53	[M − H]^−^	C_21_H_20_O_11_	447.0922	447.0929	1.57	Isoorientin	447.0929, 429.0821, 357.0613, 327.0508, 285.0420, 133.0281	[27]
**36**	8.55	[M − H]^−^	C_8_H_14_O_4_	173.0808	173.0808	0.00	Suberic acid	173.0808, 129.0906, 111.0800	[28]
**37**	8.59	[M + H]^+^	C_10_H_8_O_4_	193.0495	193.0490	−2.95	Isoscopoletin	193.0490, 178.0255, 133.0280	Standard
**38**	8.60	[M − H]^−^	C_26_H_32_O_12_	535.1810	535.1818	1.49	1-Hydroxylpinoresinol 4’-O-glucopyranoside	535.1818, 373.1284, 343.1182	[19]
**39**	8.62	[M + H]^+^	C_21_H_20_O_12_	465.1027	465.1024	−0.65	Hyperoside	465.1024, 303.0494, 257.0439, 229.0493, 153.0181	[29]
	8.62	[M − H]^−^	C_21_H_20_O_12_	463.0871	463.0882	2.38	Hyperoside	463.0882, 301.0350, 271.0244, 178.9974, 151.0023	[29]
**40**	8.77	[M − H]^−^	C_9_H_8_O_3_	163.0389	163.0390	0.61	Coumaric acid	163.0390, 119.0488	[19]
**41**	8.88	[M + H]^+^	C_16_H_12_O_7_	317.0656	317.0649	−2.21	Isorhamnetin	317.0649, 302.0416, 285.0388, 274.0467, 153.0180	[30]
**42**	8.91	[M + H]^+^	C_15_H_10_O_6_	287.0550	287.0545	−1.74	Kaempferol	287.0545, 269.0437, 231.0651, 213.0541, 153.0180, 121.0285	[29]
**43**	8.92	[M − H]^−^	C_22_H_22_O_12_	477.1028	477.1034	1.26	Isorhamnetin-3-O-glucoside isomer	477.1034, 314.0430, 285.0402, 271.0247, 243.0295	[22]
**44**	8.94	[M + H]^+^	C_10_H_8_O_4_	193.0495	193.0493	−1.04	Scopoletin	193.0493, 178.0258, 175.1479, 150.0309, 133.0283, 109.0857	Standard
	8.94	[M − H]^−^	C_10_H_8_O_4_	191.0339	191.0340	0.52	Scopoletin	191.0340, 176.0105, 146.973,111.0074, 102.9474	
**45**	8.96	[M − H]^−^	C_21_H_20_O_11_	447.0933	447.0930	−0.67	Luteoloside	447.0930, 285.0420, 255.0296, 241.0500, 217.05.01, 227.0343, 199.0395, 151.0023	[19]
**46**	8.98	[M − H]^−^	C_10_H_10_O_4_	193.0495	193.0495	0.00	Ferulic acid	193.0495, 178.0261, 149.0595,134.0360, 121.0282	Standard
**47**	9.03	[M + H]^+^	C_21_H_18_O_11_	447.0922	447.0924	0.45	Apigenin-7-O-glucuronide	447.0924, 271.0595, 231.1145, 199.2475, 153.0180, 119.0490	[28]
	9.03	[M − H]^−^	C_21_H_18_O_11_	445.0765	445.0752	−2.92	Apigenin-7-O-glucuronide	445.0752, 269.0452, 175.0233, 113.0229	
**48**	9.06	[M − H]^−^	C_21_H_18_O_12_	461.0714	461.0727	2.82	Luteolin-7-O-glucuronide	461.0727, 285.0402, 151.0022, 133.0279	[31]
**49**	9.10	[M − H]^−^	C_26_H_32_O_11_	519.1861	519.1873	2.31	Matairesinoside	519.1972, 357.1340, 342.1098, 313.1464, 221.0804, 161.0595	[19]
**50**	9.22	[M − H]^−^	C_9_H_16_O_4_	187.0964	187.0966	1.07	Azelaic acid	187.0966, 125.0958, 97.0644	[32]
**51**	9.31	[M − H]^−^	C_9_H_10_O_3_	165.0546	165.0545	−0.61	*p*-Hydroxybenzene propanoic acid	165.0545, 147.0439, 136.9310, 119.0488, 72.9916	[33]
**52**	9.33	[M − H]^−^	C_20_H_22_O_7_	373.1282	373.1289	1.88	1-Hydroxylpinoresinol	373.1289, 343.1176, 313.1081, 298.0844, 147.0439, 123.0074, 109.0277	[19]
**53**	9.68	[M − H]^−^	C_7_H_6_O_3_	137.0233	137.0232	−0.73	Salicylic acid	137.0232, 93.0331	Standard
**54**	9.82	[M + H]^+^	C_11_H_10_O_4_	207.0652	207.0649	−1.45	Scoparone	207.0649, 191.0337, 163.0388, 151.0752, 121.0648, 105.0703	Standard
**55**	10.28	[M + H]^+^	C_15_H_10_O_6_	287.0550	287.0545	−1.74	Luteolin	287.0545, 153.0180, 131.0439	Standard
	10.28	[M − H]^−^	C_15_H_10_O_6_	285.0393	285.0403	3.51	Luteolin	285.0403, 175.0387, 133.0281,121.0279, 107.0125, 83.0124	
**56**	10.32	[M − H]^−^	C_15_H_10_O_7_	301.0343	301.0355	3.99	Quercetin	301.0355, 229.0504, 201.0565, 178.9975, 151.0024, 121.0281	Standard
**57**	10.83	[M − H]^−^	C_20_H_22_O_6_	357.1332	357.1341	2.52	Matairesinol	357.1341, 342.1097, 313.0370, 283.0078,	[19]
**58**	11.06	[M + H]^+^	C_15_H_10_O_5_	271.0601	271.0595	−2.21	Apigenin	271.0595, 153.0181, 119.0492	[26]
	11.06	[M − H]^−^	C_15_H_10_O_5_	269.0444	269.0453	3.35	Apigenin	269.0453, 251.0590, 227.0341, 181.0644, 151.0025, 117.0332	
**59**	11.16	[M − H]^−^	C_16_H_12_O_6_	299.0550	299.0560	3.34	Chrysoeriol	299.0560, 284.0326, 256.0372, 255.0293, 227.0345	[31]
**60**	11.19	[M − H]^−^	C_15_H_10_O_6_	285.0394	285.0403	3.16	7-Hydroxy-emodin	285.0403, 257.0449, 211.0380	[34]
**61**	11.41	[M − H]^−^	C_16_H_12_O_6_	299.0550	299.0561	3.68	Diosmetin	299.0561, 284.0325, 256.0378, 255.0296, 227.0344	[31]
**62**	11.51	[M − H]^−^	C_16_H_12_O_7_	315.0499	315.0508	2.86	Rhamnetin	315.0508, 300.0271, 151.0024,107.0123	[35]
**63**	11.67	[M − H]^−^	C_16_H_12_O_5_	283.0601	283.0611	3.53	1-O-Methyl-emodin	283.0611, 268.0376, 239.0346, 211.0395	[34]
**64**	12.75	[M − H]^−^	C_15_H_12_O_4_	255.0651	255.0659	3.14	Pinocembrin	255.0659, 213.0549, 171.0441, 151.0024	[36]
**65**	12.95	[M + H]^+^	C_17_H_14_O_6_	315.0863	315.0857	−1.90	7,3′-Dihydroxy-8,4′-dimethoxyisoflavone/isomer	315.0857, 300.0623	[37]
**66**	13.21	[M − H]^−^	C_16_H_12_O_5_	283.0601	283.0610	3.18	Physcion	283.0610, 268.0377, 239.0346, 211.0393	[34]
**67**	13.77	[M + H]^+^	C_18_H_39_NO_3_	318.3002	318.2996	−1.89	2-Amino-1,3,4-octadecanetriol	318.2996, 300.2891, 282.2787, 264.2681, 60.0452	[38]
**68**	14.02	[M − H]^−^	C_15_H_10_O_5_	269.0444	269.0455	4.09	Emodin	269.0455, 241.0504, 225.0552, 210.0320	Standard
**69**	17.25	[M − H]^−^	C_30_H_48_O_3_	455.3531	455.3528	−0.66	Oleanic acid	455.3528, 240.9500, 206.1664, 82.4031	Standard

## Data Availability

The data presented in this study are available on request from the corresponding author.

## Supplementary Materials

The following supporting information can be downloaded at: https://www.mdpi.com/article/10.3390/molecules27103299/s1. Figure S1: Product ion spectra of standard samples of isoscopoletin (A), scopoletin (B), and scoparone (C) in positive ionization mode. Figure S2: Product ion spectra of standard samples of protocatechuic acid (A), aesculetin (B), caffeic acid (C), ferulic acid (D), salicylic acid (E), luteolin (F), quercetin (G), emodin (H) and oleanic acid (I) in negative ionization mode. Figure S3: Chemical structures of 69 compounds identified in the 80% methanol extract of RS.

## Sample Availability

Samples of the compounds are not available from the authors.
