# Supplementary material for: Chemical Constituent Analysis of Ranunculus sceleratus L. Using Ultra-High-Performance Liquid Chromatography Coupled with Quadrupole-Orbitrap High-Resolution Mass Spectrometry
Source: Molecules. 2022 May 20;27(10):3299. doi: 10.3390/molecules27103299 (PMC9145087; doi:10.3390/molecules27103299)
Supplement: Supplementary file 1 [file molecules-27-03299-s001.zip › molecules-1734346-supplementary.pdf]

- Supplementary Material -

Chemical Constituent Analysis of *Ranunculus sceleratus* L. Using Ultra-High-Performance Liquid Chromatography Coupled with Quadrupole-Orbitrap High-Resolution Mass Spectrometry

Shanshan Cao [1], Min Hu [1], Lingli Yang [1], Meiqin Li [1], Zhen Shi [1], Wenming Cheng [1,*], Yazhong Zhang [2], Fei Chen [1], Sheng Wang [3] and Qunlin Zhang [1,*]

1 School of Pharmacy, Anhui Medical University, Hefei 230032, China; 15555455280@163.com (S.C.); minhu3597@163.com (M.H.); 15850658857@163.com (L.Y.); lmq2860735640@163.com (M.L.); 18269712818@163.com (Z.S.); 17555801160@139.com (F.C).

2 Anhui Institute for Food and Drug Control, Hefei 230051, China; 13956985695@139.com (Y.Z.)

3 The Center for Scientific Research of Anhui Medical University, Hefei 230032, China; wangsheng_cpu@163.com (S.W.)

* Correspondence: chengwm@ahmu.edu.cn (W. C.); qlzhang@ahmu.edu.cn (Q. Z.)

Table of Contents

Figure S1: Product ion spectra of standard samples of isoscopoletin (A), scopoletin (B) and scoparone (C) in positive ionization mode.

Figure S2: Product ion spectra of standard samples of protocatechuic acid (A), aesculetin (B), caffeic acid (C), ferulic acid (D), salicylic acid (E), luteolin (F), quercetin (G), emodin (H) and oleanic acid (I) in negative ionization mode.

Figure S3: Chemical structures of 69 compounds identified in the 80% methanol extract of RS.

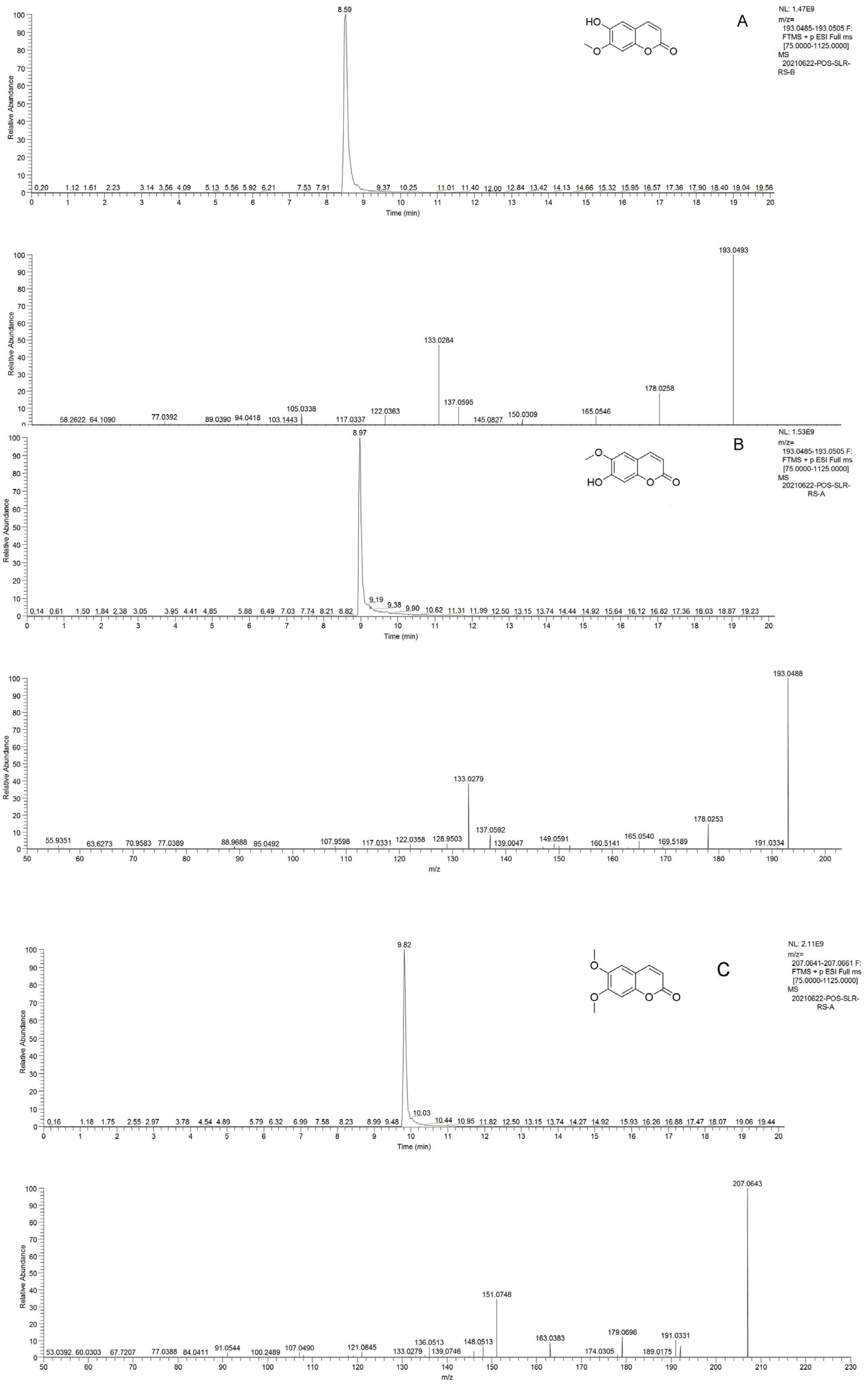

Figure S1. Product ion spectra of standard samples of isoscopoletin (A), scopoletin (B) and scoparone (C) in positive ionization mode.

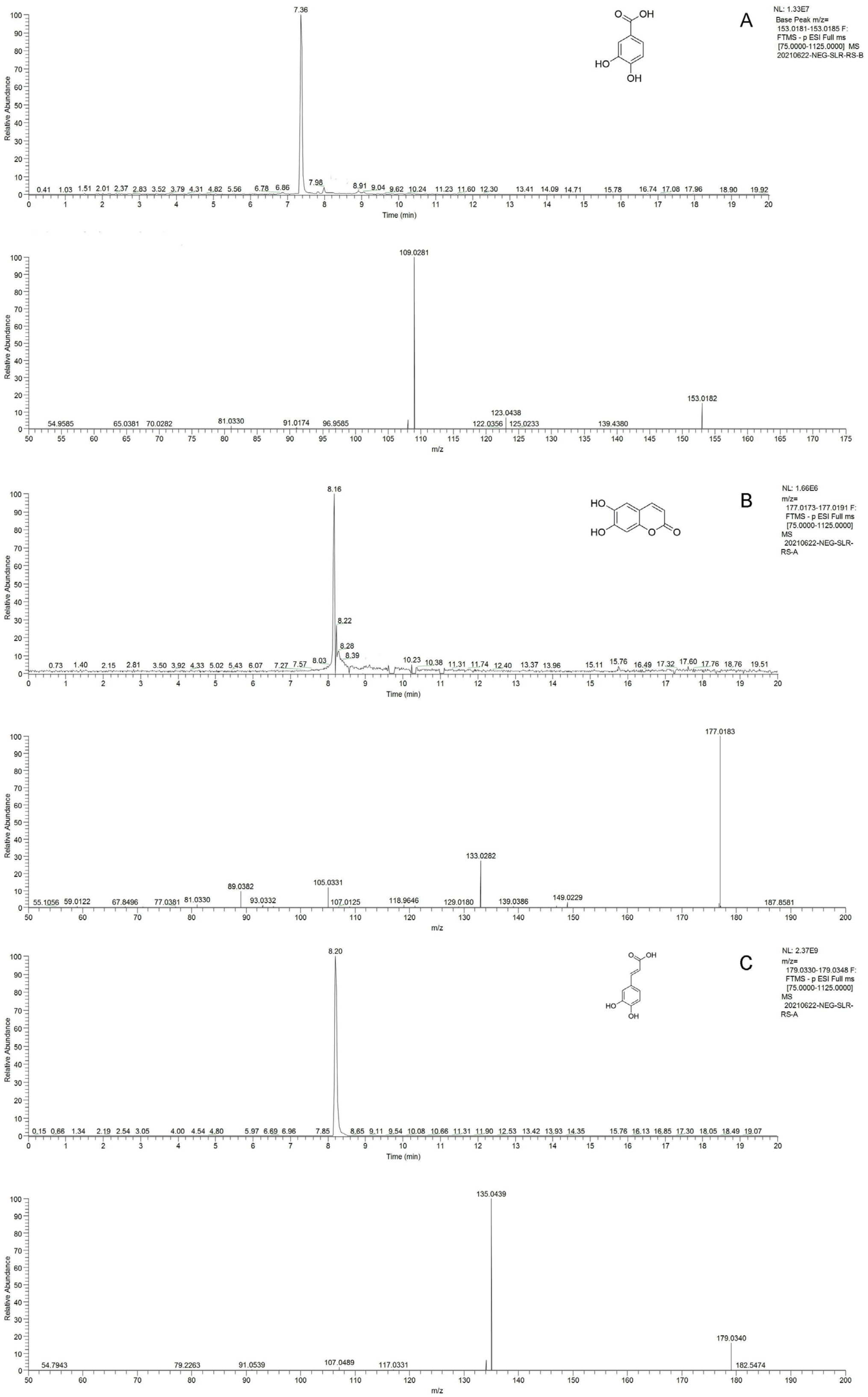
A
NL: 1.33E7
Base Peak m/z= 153.0181-153.0185 F: FTMS - p ESI Full ms [75.0000-1125.0000] MS 20210622-NEG-SLR-RS-B
Relative Abundance
Time (min)
7.36
109.0281
153.0182
m/z
B
NL: 1.66E6
m/z= 177.0173-177.0191 F: FTMS - p ESI Full ms [75.0000-1125.0000] MS 20210622-NEG-SLR-RS-A
8.16
177.0183
133.0282
C
NL: 2.37E9
m/z= 179.0330-179.0348 F: FTMS - p ESI Full ms [75.0000-1125.0000] MS 20210622-NEG-SLR-RS-A
8.20
135.0439
179.0340
HO
OH
O

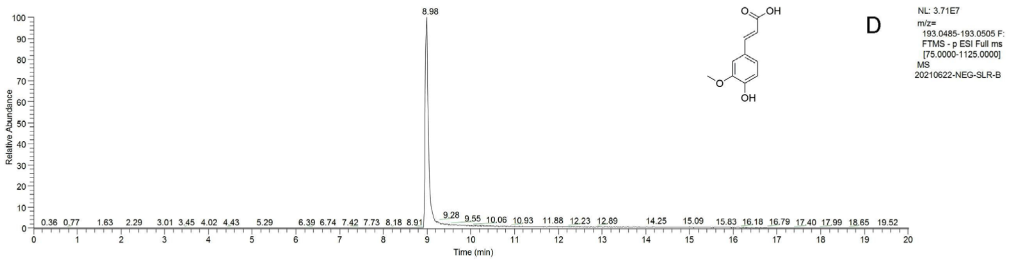
D
NL: 3.71E7
m/z= 193.0485-193.0505 F: FTMS - p ESI Full ms [75.0000-1125.0000] MS 20210622-NEG-SLR-B
Relative Abundance
Time (min)
8.98

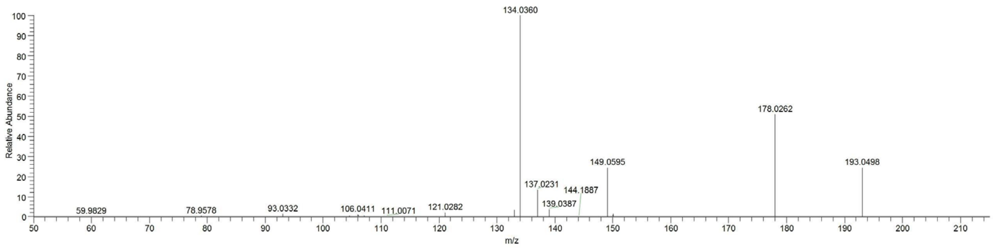
Relative Abundance
m/z
134.0360
178.0262
193.0498
149.0595
137.0231
144.1887
139.0387
121.0282
106.0411
111.0071
93.0332
78.9578
59.9829

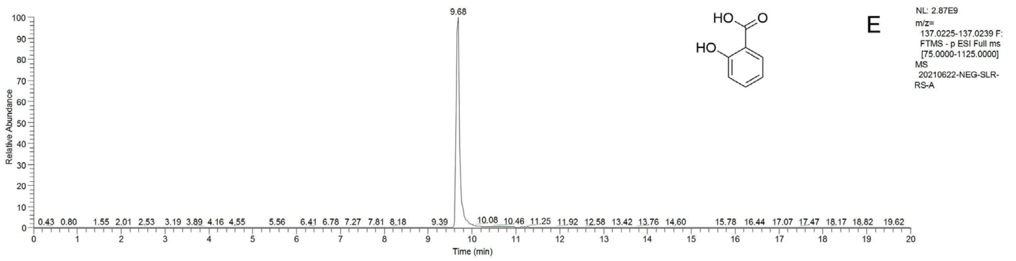
E
NL: 2.87E9
m/z= 137.0225-137.0239 F: FTMS - p ESI Full ms [75.0000-1125.0000] MS 20210622-NEG-SLR-RS-A
Relative Abundance
Time (min)
9.68

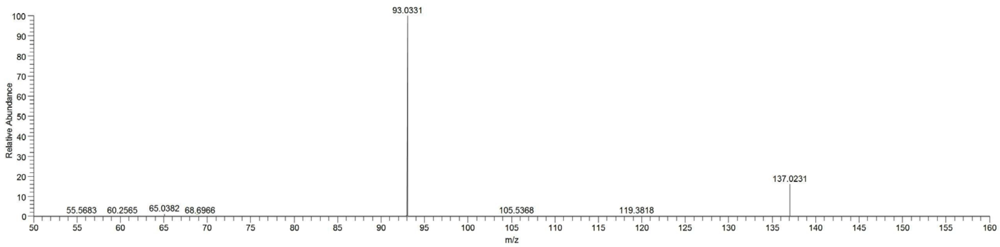
Relative Abundance
m/z
93.0331
137.0231
55.5683
60.2565
65.0382
68.6966
105.5368
119.3818

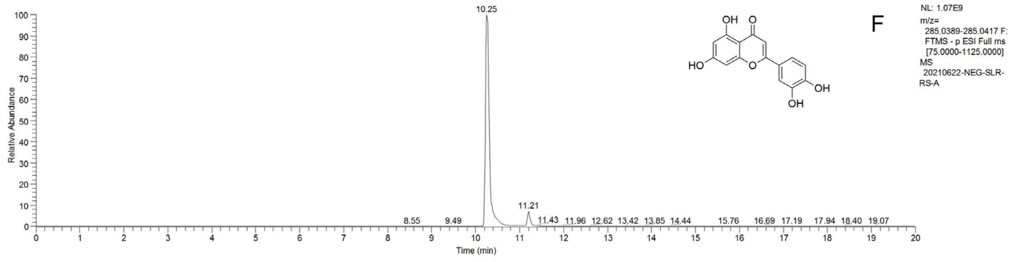
F
NL: 1.07E9
m/z= 285.0389-285.0417 F: FTMS - p ESI Full ms [75.0000-1125.0000] MS 20210622-NEG-SLR-RS-A
Relative Abundance
Time (min)
10.25
11.21

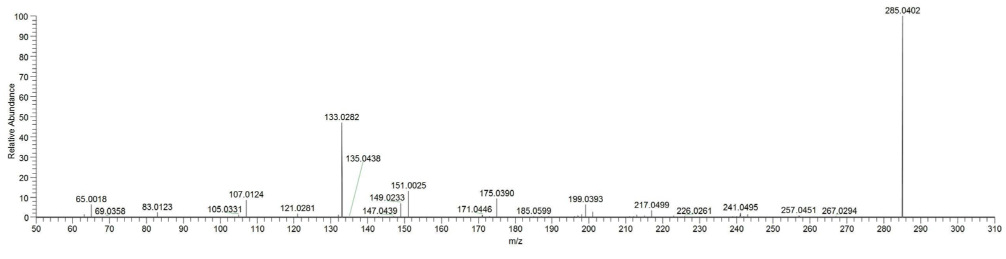
Relative Abundance
m/z
285.0402
133.0282
135.0438
151.0025
149.0233
147.0439
107.0124
105.0331
121.0281
65.0018
69.0358
83.0123
175.0390
171.0446
185.0599
199.0393
217.0499
226.0261
241.0495
257.0451
267.0294

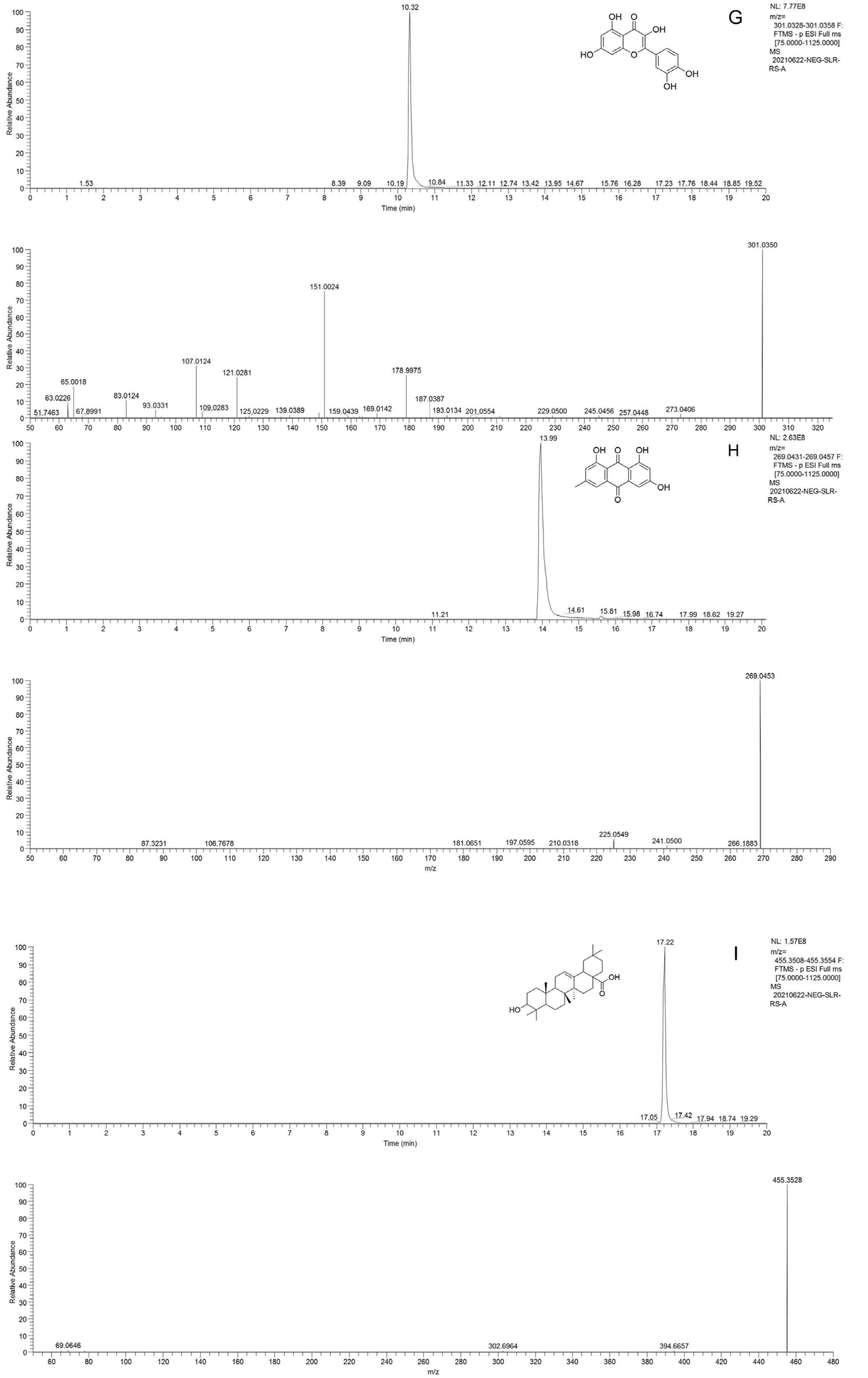

Figure S2. Product ion spectra of standard samples of protocatechuic acid (A), aesculetin (B), caffeic acid (C), ferulic acid (D), salicylic acid (E), luteolin (F), quercetin (G), emodin (H) and oleanic acid (I) in negative ionization mode.

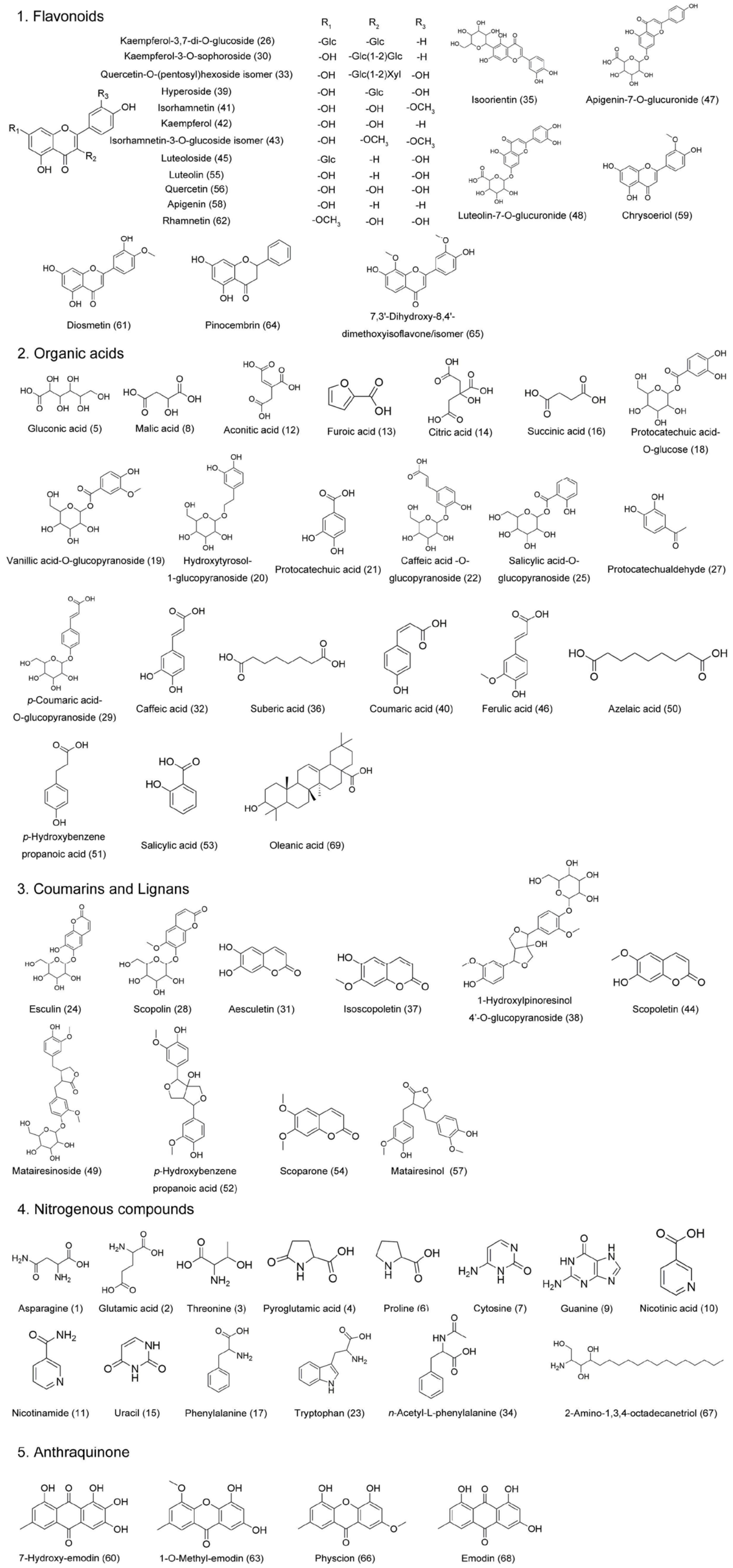

Figure S3. Chemical structures of 69 compounds identified in the 80% methanol extract of RS.
